# Probability of dengue transmission and propagation in a non-endemic temperate area: conceptual model and decision risk levels for early alert, prevention and control

**DOI:** 10.1186/s13071-018-3280-z

**Published:** 2019-01-16

**Authors:** Cecilia A. Marques-Toledo, Maria Mercedes Bendati, Claudia T. Codeço, Mauro M. Teixeira

**Affiliations:** 1 Departamento de Bioquimica e Imunologia do Instituto de Ciencias Biologicas, Universidade Federal de Minas Gerais, Belo Horizonte, Brazil; 2 Vigilancia de Roedores e Vetores da Secretaria Municipal de Saude (CGVS/SMS), Porto Alegre, Brazil; 3 Programa de Computacao Cientifica, Fundacao Oswaldo Cruz, Rio de Janeiro, Brazil

**Keywords:** Dengue, Transmission, Risk model, Non-endemic

## Abstract

**Background:**

Dengue viruses have spread rapidly across tropical regions of the world in recent decades. Today, dengue transmission is observed in the Americas, Southeast Asia, Western Pacific, Africa and in non-endemic areas of the USA and Europe. Dengue is responsible for 16% of travel-related febrile illnesses. Although most prevalent in tropical areas, risk maps indicate that subtropical regions are suitable for transmission. Dengue-control programs in these regions should focus on minimizing virus importation, community engagement, improved vector surveillance and control.

**Results:**

We developed a conceptual model for the probability of local introduction and propagation of dengue, comprising disease vulnerability and receptivity, in a temperate area, considering risk factors and social media indicators. Using a rich data set from a temperate area in the south of Brazil (where there is active surveillance of mosquitoes, viruses and human cases), we used a conceptual model as a framework to build two probabilistic models to estimate the probability of initiation and propagation of local dengue transmission. The final models estimated with good accuracy the probabilities of local transmission and propagation, with three and four weeks in advance, respectively. Vulnerability indicators (number of imported cases and dengue virus circulation in mosquitoes) and a receptivity indicator (vector abundance) could be optimally integrated with tweets and temperature data to estimate probability of early local dengue transmission.

**Conclusions:**

We demonstrated how vulnerability and receptivity indicators can be integrated into probabilistic models to estimate initiation and propagation of dengue transmission. The models successfully estimate disease risk in different scenarios and periods of the year. We propose a decision model with three different risk levels to assist in the planning of prevention and control measures in temperate regions at risk of dengue introduction.

**Electronic supplementary material:**

The online version of this article (10.1186/s13071-018-3280-z) contains supplementary material, which is available to authorized users.

## Background

Dengue is one of the world’s most important neglected tropical diseases [1]; its incidence and the risk of epidemics have increased > 30-fold in recent decades, alongside the geographical expansion of *Aedes aegypti*, the major vector for dengue virus (DENV) transmission [2, 3]. It is estimated that there are approximately 390 million dengue infections each year, of which 96 million are thought to manifest clinically [4]. Dengue transmission is observed in the Eastern Mediterranean, the Americas, Southeast Asia, Western Pacific and Africa. There are frequent cases in non-endemic areas, including the USA and Europe [1, 5]. In these areas, dengue is responsible for an increasing number of travel-related acute febrile illnesses [6]. Travel-related illnesses are reported in 20–70% of subjects returning from tropical to temperate countries [7]; more severe and potentially fatal forms of dengue infection have been reported in up to 16% of those cases [8]. The currently available vaccine is only partially effective and not recommended for dengue-naive individuals [9]. In addition, there is an increasing incidence and geographical expansion of dengue transmission, increasing the socioeconomic burden, whilst current control strategies are ineffective [9, 10]. New risk maps and infection estimates provide novel insights into future risk under scenarios of socioeconomic and environmental change [4]. The surveillance of vulnerability and receptivity risk factors together with dengue notification data can provide more accurate data to inform the prioritization of research, health policies and financial resources towards dengue control [11]. Efforts to improve dengue surveillance in non-endemic areas are especially important during mass gatherings (sports, cultural and religious events) due to the growth of the global population and the global mobility of people infected with diseases [12].

According to official records, dengue was introduced to Brazil in the 1980s [13]. Since then, it has been characterized by geographical spread and an increase in the incidence of reported cases [14]. As an exception, the subtropical southern region of Brazil has not yet established stable local transmission of the disease, despite the presence of the vector [15]. In Porto Alegre, the largest city of the southernmost state, the first record of an imported dengue case occurred in 2002 [16], but local transmission was confirmed only in 2010 [17]. Even though the number of dengue cases in the southern region of Brazil has increased in recent years, it is much lower than other regions of the country [17], mostly because of the local temperate climate [18]. There are cold winters, with the temperature ranging from -3 ºC to 18 °C, which are less suitable for mosquito reproduction [17]. Several countries or regions in the northern hemisphere (including Florida in the USA, southern Italy, France and Spain) have similar weather patterns and risk factors for dengue introduction, including the proximity to endemic regions, presence of competent vectors, nonimmune population, and lack of citizen engagement [19]. The city of Porto Alegre has experienced several episodes of dengue introduction in the last few years and has developed an integrated surveillance and prevention protocol that includes entomological, virological and active epidemiological components.

Here we used this rich dataset to develop probabilistic models for the local transmission of dengue in a non-endemic city. We showed how vulnerability and receptivity risk factors can be used to estimate the local probability of dengue transmission. We developed probabilistic models to estimate initiation and propagation of local dengue transmission, three and four weeks in advance, respectively. We propose a decision model with three different risk levels, based on probability of disease occurrence, to assist the planning of prevention and control measures in temperate regions at risk of dengue introduction.

## Methods

### Study area

Porto Alegre (0°01'40"S, 51°13'43"W) is the main city of the Brazilian southernmost state, Rio Grande do Sul. The city has an area of 496.68 km^2^, an estimated population of 1,409,351 inhabitants and a high human development index of 0.81 [20]. The climate is classified as subtropical humid [18].

### Seroprevalence survey

To determine the level of exposure to dengue of the population of Porto Alegre, we performed a blood bank seroprevalence evaluation of healthy blood donors. Samples were collected at Hemocentro do Rio Grande do Sul (Hemorgs) Blood Bank Center, from July to September 2015. Samples were collected in separation gel tubes, stored and transported at -20 °C to the Immunopharmacology Laboratory at the Federal University of Minas Gerais (UFMG). These samples were analyzed by dengue IgG Capture enzyme-linked immunosorbent assay (ELISA), following instructions from the manufacturer (EuroImmun, Luebeck, Germany). Seroprevalence was estimated by the point prevalence method [21] with 95% confidence intervals.

### Dengue virus (DENV) monitoring in mosquitoes

To identify which dengue viruses were circulating in the mosquito population, we evaluated virus presence in adult (male and female) *Aedes aegypti* mosquitoes captured by the adult trap, MosquiTRAP part of the entomological surveillance program of the city, described below [16]. Mosquitoes from traps were collected weekly and stored in tubes containing nucleic acid conservation solution, guanidine thiocyanate (250 μl, 1.5%). Each tube contained mosquitoes from the same trap (mean = 2 mosquitoes). For the analysis, mosquitoes were macerated using zirconium microspheres for 20 s in a FastPrep-24 (MPBiomedicals, Santa Ana, CA, USA), then centrifuged at 10,000× *rpm* (5 min). To extract the viral RNA, the supernatant (20 μl) of samples was pooled into groups of 20 mosquitoes. RNA extracts were obtained using the BioGene Viral DNA/RNA Extraction Kit (Bioclin, Belo Horizonte, Brazil). One-step RT-PCR was optimized and carried out using an iTaq Universal Probes One-Step Kit (Bio-Rad Laboratories, Hercules, CA, USA) in a QuantStudio 5 (Applied Biosystems, Waltham, USA) system. The one-step RT-PCR consisted of a 20 min reverse transcription step at 50 °C and then 2 min of Taq-polymerase activation at 95 °C, followed by 45 cycles of PCR at 95 °C for 15 s and 55 °C for 1 min. The primers and probe sequences used in these analyses have been described previously [22]. Samples were considered positive if the fluorescence curve was equal or higher than those of positive controls. Supernatants from cultures of Vero cells infected with dengue were used as positive controls.

### Secondary data sources

#### Official dengue case data

Dengue data were obtained from the system for disease notification held by the Brazilian Ministry of Health (Sinannet). All suspected dengue cases notified by hospitals and health local units were tested by immunological ELISA tests (IgM and NS1) performed at the local accredited laboratory [16]. We considered the date of first symptoms as the date of the disease, to aggregate the confirmed cases per week, between September 2012 and December 2017. Confirmed cases were classified as imported or local based on the reported epidemiological investigation, which defined as imported those patients with travel history to dengue endemic areas in the 10 days prior to first symptoms (which assumes a 10-day-long intrinsic incubation period) [2]. Local cases were considered as those patients that have acquired the virus inside the city.

#### Climate data

Daily rainfall, temperature (minimum, average and maximum), and average relative air humidity data were obtained from the Brazilian National Institute of Meteorology (INMET) [20]. The daily data were averaged per week.

#### Mosquito data

The routine larval surveys carried out in the city provided two entomological indices: house index (number of positive containers/total number of houses inspected) and Breteau index (number of larvae positive containers/total number of containers found) [23]. Another source of mosquito data is based on the collection of adult *Aedes aegypti* mosquito by the MI-Aedes system, using the sticky trap MosquiTRAP (Ecovec LTDA, Belo Horizonte, Brazil) [24, 25]. The surveillance system had 935 sticky traps distributed in 29 of the 80 neighborhoods (44% of the study area), at a separation of 250 m, as described previously [26]. The traps were inspected weekly. Data for this study encompasses 234 weeks, from September 2012 to March 2017. The entomological index provided by the system was the Index of mean number of female *Ae. aegypti* (IMFA, total number of female *Ae. aegypti* captured divided by the number of inspected traps).

#### Tweets

Twitter messages (tweets) related to dengue personal experiences were obtained from the web-application www.observatorio.inweb.org.br/dengue/ [27]. Tweets from September 2012 to December 2017, geolocated to Porto Alegre, were aggregated per week. Tweets were collected by application through a special access (api), which extracts all the tweets with the key words: “dengue”, “*Aedes*” and “*aegypti*” [27]. Tweets were classified into four categories: personal experience, parody, opinion, information and marketing campaigns. Here we analyzed only the number of tweets classified as personal experience of dengue which was previously demonstrated to be highly associated to dengue incidence [27, 28].

### Data analyses

#### Exploratory analyses

Evidence of association between the response variable “number of (confirmed) local dengue cases”, and the explanatory variables was assessed by linear correlation, scatterplots and autocorrelation graphs. The best time periods of lag (difference between the week of the response measure and the week of the explanatory variables measure) for each variable were selected based on autocorrelation plots (Additional file 1: Figure S1). The covariates were: number of (confirmed) imported cases of dengue; mosquito infestation index (IMFA); presence of dengue virus in mosquitoes; number of tweets with dengue content; minimum, maximum and mean temperature; and humidity and precipitation. All data were aggregated per week.

#### Conceptual model

**Fig. 1:**
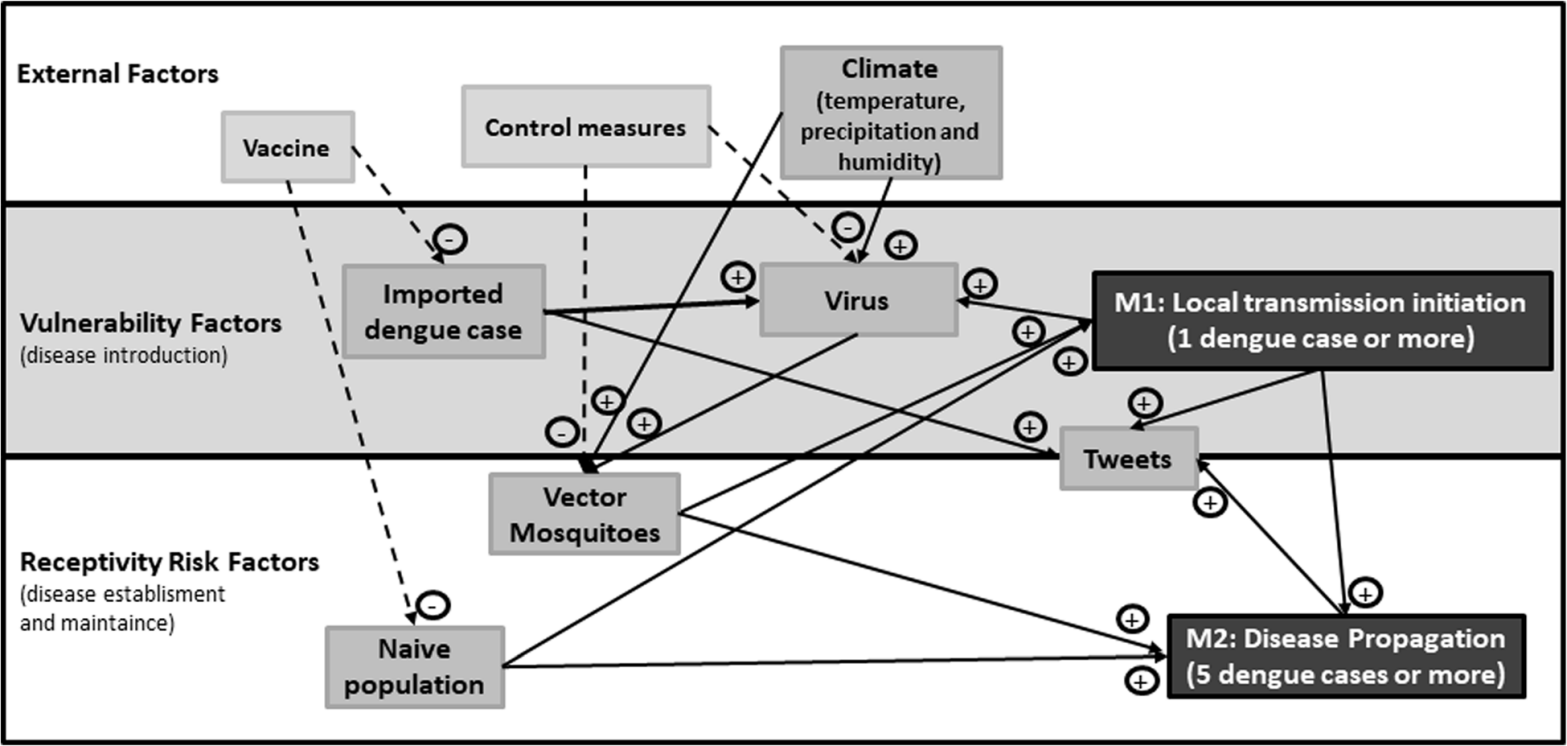
Interaction between risk factors of dengue local transmission initiation (M1) and propagation (M2)

Figure 1 shows the conceptual model describing the factors that modulate the probability of local transmission. The arrival of one or more imported cases and exposure to competent mosquitoes increase the probability of observing at least one event of local dengue transmission (the outcome variable of the Model M1). Mosquito competence is further influenced by climate and control measures. Furthermore, the occurrence of at least 5 cases of locally acquired dengue in a week was considered a marker of “dengue propagation”, and this outcome was investigated in Model M2. This threshold of 5 cases for dengue propagation was defined using the moving epidemic method (MEM), detailed below [29]. Twitter, though not a risk factor for the disease, is included in the model because it is a byproduct of the dengue activity and, as such, can be used as a proxy improving the predictive capacity of dengue incidence models, as demonstrated previously [27, 28]. We built a conceptual model for the interaction between factors that influence dengue transmission (Fig. 1). Here we divided dengue transmission probability in two different outcomes: transmission initiation (Model M1) and propagation (Model M2). The transmission initiation model was used to explain the chance of at least one dengue local case occurring in a week, while the propagation model explains the chance of this local transmission spreading to more than five cases.

#### Estimation of epidemic threshold

The number of cases used as a threshold for declaring dengue propagation was defined using the moving epidemic method (MEM). MEM evaluates the distribution of cases per week from a time series of dengue notifications and identifies the average length, start and end week of dengue seasons looking at the percentiles of the distribution. MEM splits the season in three periods (pre-epidemic, epidemic and post-epidemic). The baseline and epidemic thresholds are calculated as the number of cases in the transition between pre-epidemic and epidemic periods [29] (Additional file 2: Figure S2).

#### Estimation of time-dependent reproduction numbers (R_t_)

To identify weeks with a significant increase of cases, we calculated the time-dependent reproduction number of dengue using the R0 package [30]. The generation period was considered as a gamma distribution with a mean of 2.24 weeks (SD 0.3), as described previously [31]. A sequence of weeks with *R*_*t*_ > 1 (above one) is indicative of sustainable transmission.

#### Model fitting

The probability of dengue transmission initiation (M1) and propagation (M2) were investigated using logistic regression models. The outcome of M1 was a week with one or more dengue cases (yes/no) while the outcome of M2 was a week with five or more dengue cases (yes/no). The analysis included all 275 weeks of study period, from September 2012, to March 2017. For each of the outcome variables, we started by fitting logistic regression models with each covariate at a time, testing different time lags (from 0 to 4 weeks) (Additional file 3: Table S1, Additional file 4: Table S2). The best lag was chosen using Akaike information criterion (AIC) [32], as detailed in Additional file 3: Table S1), and Additional file 1: Figure S1 and Additional file 2: Figure S2. Then, multiple regression models were fitted using a subset or all chosen lagged covariates and the best models were chosen based on AIC. To validate our model, the data were divided into training and testing (out-of-sample) sets, where training data (weeks 1 to 234) were used to fit the model and test data (weeks 235 to 275) were used to evaluate the goodness of fit of our model in an out-of-sample data set.

#### Decision model

For decision making, it is useful to have a model that accurately discriminates weeks with high and low risk of dengue transmission. The logistic models M1 and M2 estimate the probability of observing local transmission (M1) or propagation (M2) of dengue conditioned on the covariates observed 3 to 4 weeks in advance. To convert these probabilities into a risk level classification (high, moderate and low risk of dengue transmission), we used the receiving operating characteristic (ROC) curve [33]. This method finds a probability threshold that maximizes both the sensitivity [true positive rate, TPR = No. of true positives/(No. of true positives + No. of false positives)] and the specificity [true negative rate, TNR = No. of true negatives/(No. of true negatives + No. of false negatives)] of a classification rule for assigning a week as high, moderate or low risk based on the observed covariates. Accuracy of the final classification was defined as the relative proportion of correct classifications (No. of true positives + No. of true negatives / Total number.).

All analyses were performed in R version 3.4.1, using the packages *MEM* [29], *R0* [30], *ROC* [33], *ggplot2* [34], *lattice* [35] and *scales* [36]. Dengue cases were geolocated and mapped using the *ggmap* package [37] in R.

## Results

**Fig. 2:**
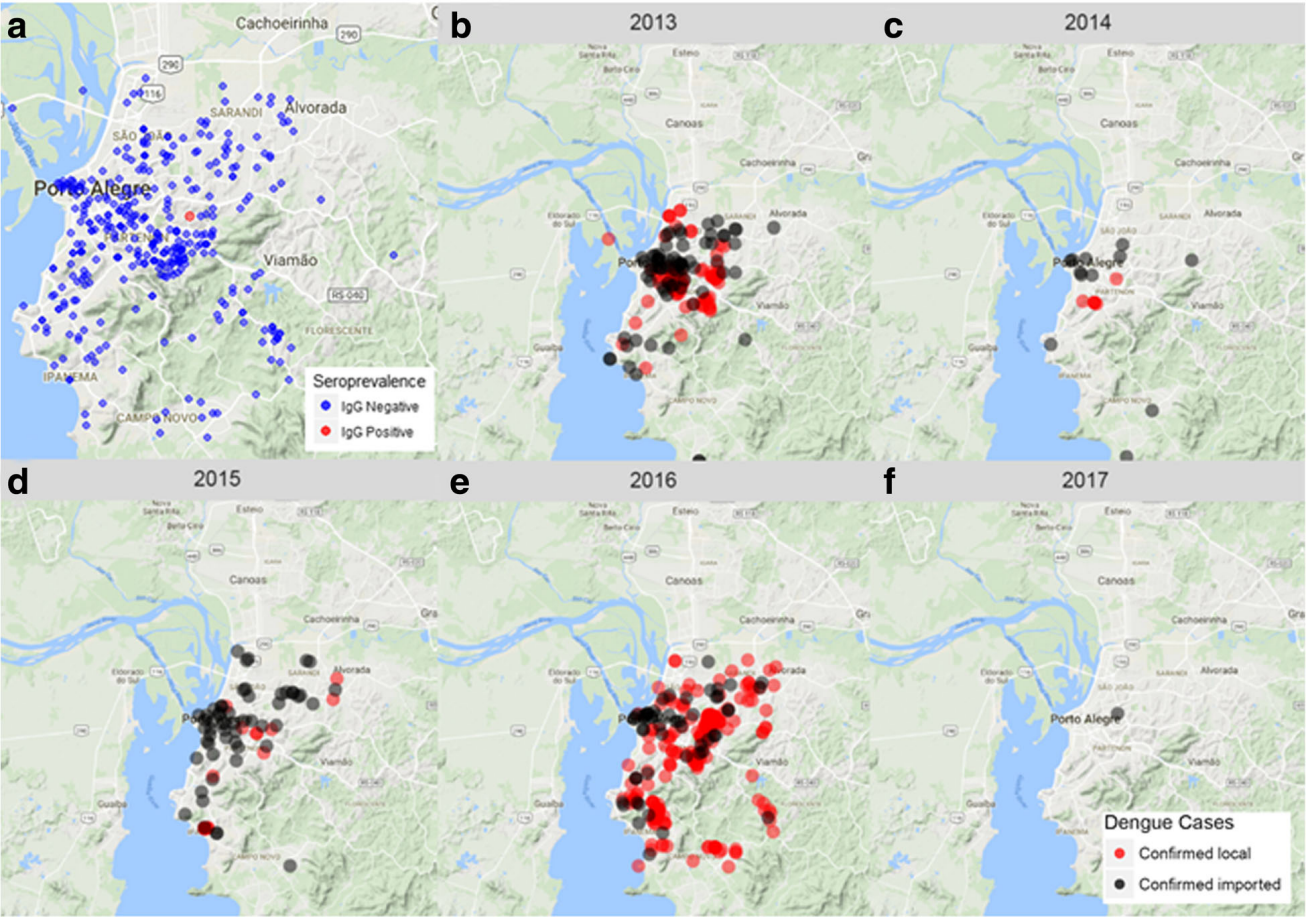
Historical dengue prevalence and incidence. **a** Dengue seroprevalence in 2015 (blue dots indicate distribution of samples collected and the red dot indicates the unique positive sample for previous dengue virus contact). **b-f** Geographical distribution of confirmed local (red) and imported (black) dengue cases in the city of Porto Alegre, from 2012 to 2017

In order to evaluate the seroprevalence for dengue in the area, we performed a blood-bank survey. We collected and evaluated 422 blood samples from healthy donors. Donors had a mean age of 36 (17–69 years) and 43.5% were female. The residency of donors was distributed throughout 71 of the 80 neighborhoods of the city (Fig. 2a). A single dengue IgG seropositive blood sample was detected from a male donor (57 years old) with residency and probable transmission at the neighborhood “Bom Jesus”, one the regions with most historical local transmission of dengue. Therefore, we estimated dengue seroprevalence in the area as 0.24% (CI: 0–1.3%), testifying the naive immunity status of the population for the disease, as was expected (Fig. 2a). Confirmed imported dengue cases were reported mostly clustered in the central area of the city (Fig. 2b, Additional file 3: Table S1), while locally acquired cases were more scattered around the city (Fig. 2b).

**Fig. 3:**
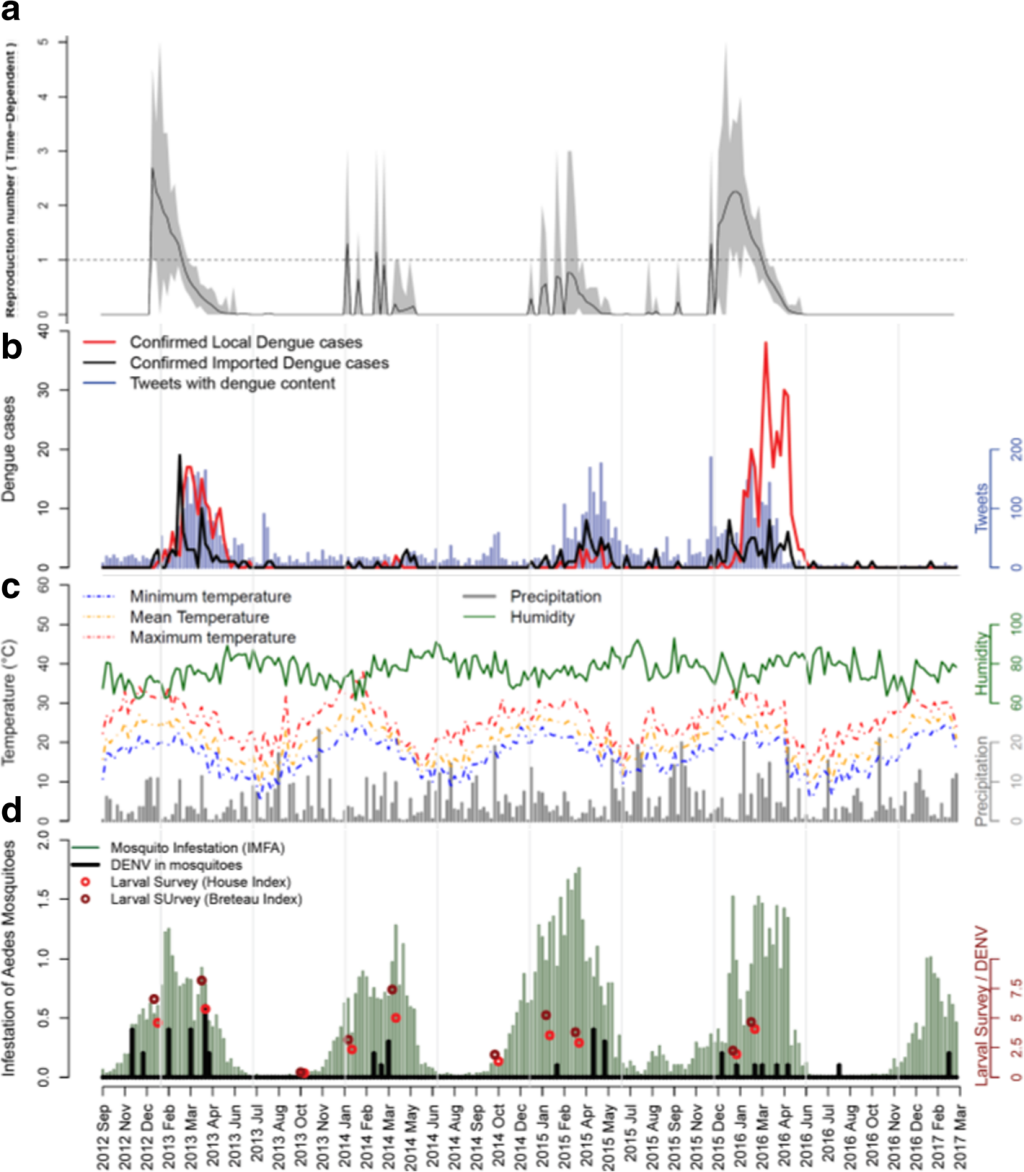
Dengue incidence and risk factor surveillance. **a** Reproduction number (time-dependent). **b** Dengue incidence of local confirmed and imported cases, and Tweets with dengue content. **c** Environmental indicators surveillance: minimum, maximum and mean temperatures; precipitation and humidity. **d** Vector indicators surveillance: house and Breteau indices of larval survey, mean infestation of adult mosquitoes (IMFA) and virus circulation in mosquitoes. All data are aggregated as weekly means from September 2012 to March 2017

The majority of imported cases came from inside the country, especially from east and southern states. Rio de Janeiro was the most visited city (24.4%). Only 10 cases (4.6%) were registered as from abroad; their origins were: Mexico (4), Paraguay (3), Colombia (1), Bolivia (1), Indonesia (1) and Costa Rica (1) (Additional file 4: Table S2). Weeks with a time-dependent reproductive number (*R*_*t*_) above one occurred in 2013 and 2016, from December to March (Fig. 3a). From the 3932 suspect cases, 697 (17.7%) were confirmed. The confirmed cases had a mean age of 37 years (range 1–89 years) and equally distributed between sexes (49.8% male). The highest number of imported cases per week was 19, while mean and maximum values of locally transmitted cases per week were 2 and 38 cases, respectively. Reports of imported dengue cases preceded local transmission of the disease (Fig. 3b). Tweets with dengue content ranged from 0 to 186 per week (Fig. 3b), with a positive correlation to dengue data. Temperatures followed a seasonal pattern with the minimum value of 4.93 °C (weekly mean) in July, and a maximum value of 38.05 °C (weekly mean) in February (Fig. 3c). Humidity and precipitation were evenly distributed throughout all years. Larval indices varied from 0.35 (HI) and 0.42 (BI) in the winter, to 5.76 (HI) and 8.17 (BI) in the summer (Fig. 3d). Indices above 3.9 are considered indicative of high risk for dengue transmission, according to the Brazilian health authorities [23]. The mosquito mean infestation index (IMFA), which is considered critical when higher than 0.6 [24], varied between 0–1.76 in this study. Dengue virus presence in mosquitoes was detected only in 20 weeks (8.5%), with up to 6 positive traps in a single week (Fig. 3d). During this period, DENV subtypes 1, 3 and 4 were detected in mosquitoes (data not shown).

### Model M1

**Fig. 4:**
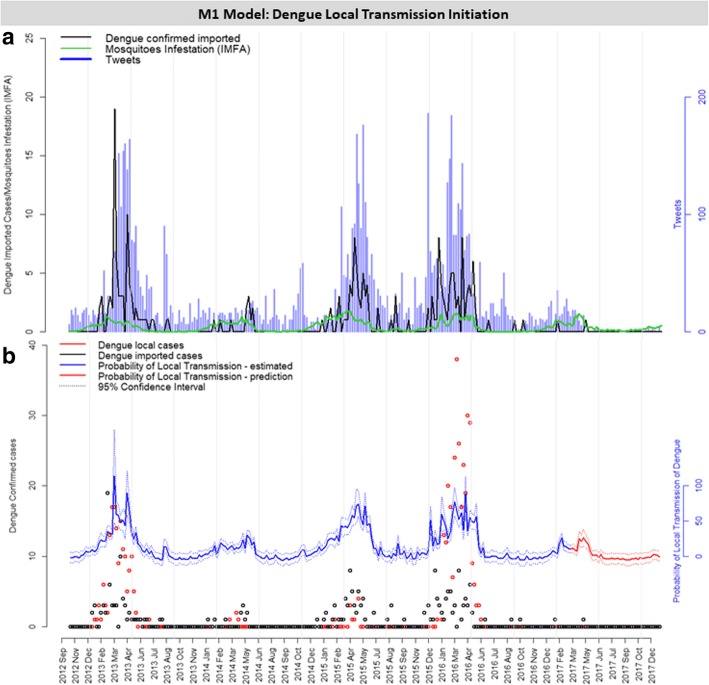
M1 model: probability risk of local dengue transmission initiation. **a** Weekly risk factors considered in Model 1 to determine the transmission probability. **b** Probability of local transmission of dengue to occur every week (red line) with 95% confidence interval (red dashed lines)

Table 1 shows the covariates associated with the outcome of at least one case of dengue occurring in a week (M1). The preliminary models, with the individual contribution of each covariate, at different time lags, are shown in Additional file 5: Figure S3 and Additional file 6: Table S3. The combination of multiple covariates into the multiple regression model strongly improved the goodness-of-fit of the probability models, based on the reduced AIC and residual deviance indices. Several models were evaluated (Additional file 6: Table S3) and Table 1 shows those considered the best and most relevant. Among them, the best model, denominated M1, contained the following variables: number of imported dengue cases, number of tweets and IMFA, all of which with a time lag of 3 weeks in relation to the outcome variable (Table 1, Fig. 4). According to model M1, an increase in one unit in imported dengue cases, tweets and IMFA corresponded to an increased chance of 1.64, 1.02, and 7.61 for local dengue cases to occur, respectively. The resulting probability of local transmission of dengue introduction is shown in Fig. 4b. This figure also shows the probability of local dengue transmission using the out-of-sample validation data (from 2017 onwards) and demonstrates the good estimation capacity of the model, despite the very few cases detected during this period (Fig. 4b).

**Table 1:** Risk factors selected for simple and multiple logistic models for the prediction of dengue local transmission introduction probability. Model M1 (bold) was selected based on the result of AIC and residual deviance

Model	Variable	Time lag (weeks)	AIC	Residual deviance	Odds	95% CI	*P*-value
Null	1	0	261.83	259.83	–	–	
Simple	Dengue imported	3	177.88	173.88	2.98	2.21–4.19	<0.001
Simple	Mosquito IMFA	3	190.94	186.94	22.82	10.01–57.86	<0.001
Simple	Tweets	3	191.32	187.32	1.04	1.03–1.05	<0.001
Simple	Minimum temperature	4	231.22	227.22	1.26	1.15–1.38	<0.001
Simple	DENV in mosquitoes	3	252.09	248.09	1.82	1.25–2.89	<0.01
Simple	Maximum temperature	0	260.18	256.18	1.06	1.00–1.13	
Simple	Mean temperature	0	260.15	256.15	1.07	1.00–1.15	
Simple	Humidity	0	263.82	259.82	1.00	0.95–1.05	
Simple	Rain	0	263.82	259.82	1.00	0.94–1.06	
**M1 multiple**	**Dengue imported (lag 3)**	**3**	**149.33**	**141.33**	**1.64**	**1.17–2.42**	**<0.01**
	**Tweets (lag 3)**				**1.02**	**1.01–1.03**	**<0.01**
	**Mosquito IMFA (lag 3)**				**7.61**	**2.87–21.74**	**<0.001**
Multiple	Dengue imported (lag 3)	3	151.00	141.00	1.62	1.16–2.39	<0.01
	DENV in mosquitoes (lag 3)				1.17	0.68–2.08	
	Tweets (lag 3)				1.02	1.01–1.03	<0.001
	Mosquito IMFA (lag 3)				7.51	2.83–21.48	<0.001
Multiple	Dengue imported (lag 3)	3	151.14	141.14	1.65	1.17–2.44	<0.001
	Tweets (lag 3)				1.02	1.01–1.04	<0.01
	Mosquito IMFA (lag 3)				6.45	1.68–27.07	<0.01
	Minimum temperature (lag 4)				1.03	0.88–1.20	
Multiple	Dengue imported (lag 3)	3	152.84	140.84	1.63	1.16–2.41	<0.01
	DENV in mosquitoes (lag 3)				1.17	0.68–2.07	
	Tweets (lag 3)				1.02	1.01–1.03	<0.01
	Mosquito IMFA (lag 3)				6.47	1.69–27.09	<0.01
	Minimum temperature (lag 4)				1.02	0.88–1.20	
Multiple	Tweets (lag 3)	3	156.59	148.59	1.02	1.01–1.04	<0.001
	Dengue imported (lag 3)				1.98	1.41–2.87	<0.001
	Minimum temperature (lag 4)				1.19	1.06–1.35	<0.01
Multiple	Tweets (lag 3)	3	156.93	150.93	1.03	1.02–1.04	<0.001
	Mosquito IMFA (lag 3)				13.46	5.50–36.37	<0.001

*Notes:* Data indicate the influence of each variable studied in the response variable: 1 or more dengue confirmed local cases (local transmission of dengue introduction). Each variable was evaluated at 5 different time points of forecast (lag zero to four). The logistic model was performed for each unique variable. The odds column indicates how each variable affect the chance of dengue local transmission to occur. Orange marked variables were selected for further analyses

*Abbreviations*: *AIC* Akaike information criterion, *CI* confidence interval, *IMFA* mean infestation of female *Aedes aegypti*, *DENV* dengue virus

### Model M2

**Fig. 5:**
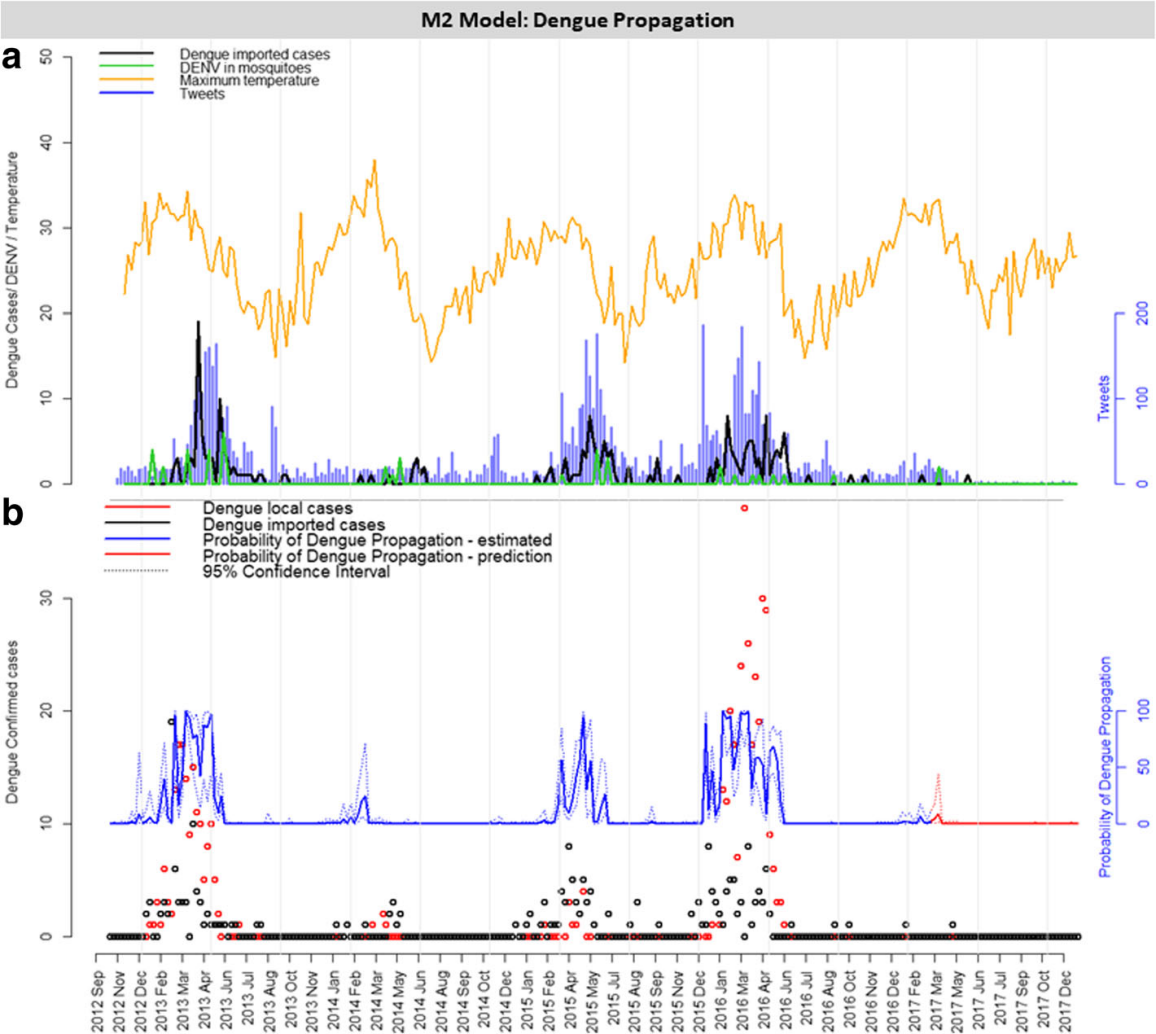
M2 model: probability risk of local dengue propagation**. a** Weekly risk factors considered to determine the probability in Model 2. **b** Probability of local transmission of dengue to propagate every week

The individual contribution of each covariate on the probability of dengue propagation is shown in Additional file 7: Figure S4. Several models were evaluated (Additional file 8: Table S4); Table 2 shows the best and most relevant models. The chosen final model, denominated M2, had the following variables: number of imported dengue cases, number of tweets, but also their interaction; DENV in mosquitoes and maximum temperature, all of them with time lag of 4 weeks in relation to the outcome variable (Table 2, Fig. 5a). According to Model M2, an increase in one unit in the number of imported dengue cases, number of tweets, number of DENV positive samples in mosquitoes and maximum temperature corresponded to an increased chance of 5.93, 1.06, 1.92 and 1.76 in the propagation of dengue cases transmission, respectively. The probability of dengue propagation, according to Model M2 is shown in Fig. 5b. As previously demonstrated, we validated the model using out-of-sample data (from 2017 onwards) with good estimation results (Fig. 5b).

**Table 2:** Risk factors selected for simple and multiple logistic models for the prediction of dengue local transmission propagation probability. Model M2 (bold) was selected based on the result of AIC and residual deviance

Model	Variable	Time lag (weeks)	AIC	Residual deviance	Odds	95% CI	*P*- value
Null	1	0	173.4	171.4	0.13	0.08–0.19	<0.001
Simple	Dengue imported	4	113.27	109.27	2.42	1.84–3.32	<0.001
Simple	Tweets	4	117.74	113.74	1.03	1.02–1.04	<0.001
Simple	Mosquito IMFA	4	137.8	133.80	13.30	5.50–35.78	<0.001
Simple	Maximum temperature	4	151.3	147.30	1.26	1.13–1.41	<0.001
Simple	DENV in mosquitoes	4	164.65	160.65	1.83	1.26–2.76	<0.01
Simple	Mean temperature	0	169.36	173.36	1.06	0.97–1.17	
Simple	Minimum temperature	0	172.77	168.77	1.08	0.98–1.19	
Simple	Humidity	0	175.37	171.37	1.00	0.94–1.07	
Simple	Rain	0	175.30	171.30	1.01	0.93–1.09	
**M2 Multiple**	**Dengue imported**	**4**	**67.19**	**55.19**	**5.93**	**2.68–17.70**	<0.001
	**Tweets**				**1.06**	**1.04–1.10**	<0.001
	**DENV in mosquitoes**				**1.92**	**1.04–4.24**	
	**Maximum temperature**				**1.76**	**1.35–2.56**	<0.001
	**Dengue imported:Tweets**				**0.99**	**0.98–0.99**	<0.01
Multiple	Dengue imported	4	69.16	55.16	5.79	2.50–17.76	<0.001
	Tweets				1.06	1.04–1.10	<0.001
	Mosquito IMFA				1.17	0.20–6.28	
	DENV in mosquitoes				1.93	1.05–4.27	
	Maximum temperature				1.75	1.34–2.56	<0.001
	Dengue imported:Tweets				0.99	0.98–0.99	<0.01
Multiple	Dengue imported	4	78.57	68.57	1.92	1.34–3.06	<0.01
	DENV in mosquitoes				1.68	0.98–3.07	
	Tweets				1.03	1.01–1.05	<0.001
	Maximum temperature				1.58	1.28–2.07	<0.001
Multiple	Dengue imported	4	79.57	67.57	1.78	1.24–2.88	<0.01
	Mosquito IMFA				2.16	0.46–9.88	
	DENV in mosquitoes				1.69	0.98–3.14	
	Tweets				1.03	1.02–1.05	<0.001
	Maximum temperature				1.54	1.24–2.03	<0.001
Multiple	Dengue imported	4	80.13	72.13	1.95	1.35–3.08	<0.01
	Tweets				1.03	1.02–1.05	<0.001
	Maximum temperature				1.52	1.25–1.94	<0.01
Multiple	Tweets	4	94.79	88.79	1.04	1.03–1.06	<0.001
	Maximum temperature				1.47	1.25–1.80	<0.001
Multiple	Dengue imported	4	97.08	89.09	1.50	1.15–2.10	<0.01
	Tweets				1.02	1.01–1.03	<0.001
	Mosquito IMFA				5.47	1.65–19.57	<0.01

*Notes:* Data indicate the influence of each variable studied in the response variable: 5 or more dengue confirmed local cases (local transmission of dengue propagation). Each variable was evaluated at 5 different time points of forecast (lag zero to four). The logistic model was performed for each unique variable. The odds column indicates how each variable affect the chance of dengue local transmission to occur

*Abbreviations*: *AIC* Akaike information criterion, *CI* confidence interval, *IMFA* mean infestation of female *Aedes aegypti*, *DENV* dengue virus

### Scenarios

**Fig. 6:**
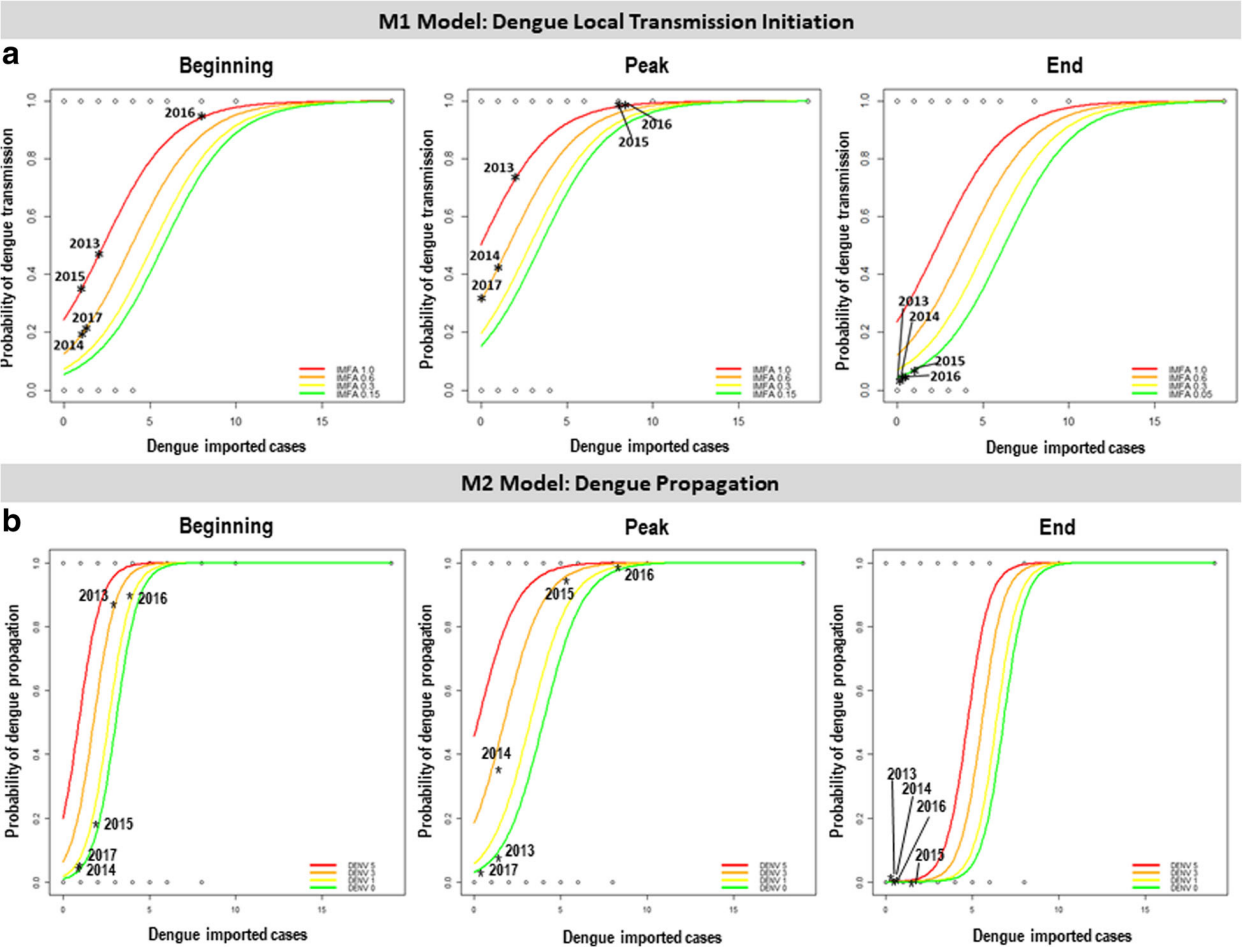
Estimated probability of dengue local transmission initiation (**a**) and propagation (**b**) in different scenarios and time periods of the year

To better visualize the effect of each covariate in the probability of local transmission and propagation of dengue, we applied the models in three specific moments of the epidemic curve: the beginning phase in January, the peak in March and the no transmission period in July (Figs. 2a, 6). The probability of transmission initiation (Model M1) in these scenarios is shown in Fig. 6a. Tweets were fixed in their mean values. The results show that detecting imported dengue cases causes an important increase in the probability of local transmission, and the presence of more mosquitoes can further increase this probability.

We also assessed the effect of imported cases and viral detection in mosquitoes on the probability of dengue propagation, according to Model M2 (Fig. 6b). Tweets and maximum temperature were fixed. Again, detecting imported dengue cases was strongly associated with an increased probability of dengue propagation, which was further increased by detecting viruses in mosquitoes (Fig. 6b). In Fig. 6, the stars indicate the estimated probabilities observed in 2012 to 2017, according to Model M2. The predictions were in accordance with the observations: 2016 was the year with highest dengue activity, and probability of local transmission and propagation (Figs. 2b, 3a).

### Decision model

**Fig. 7:**
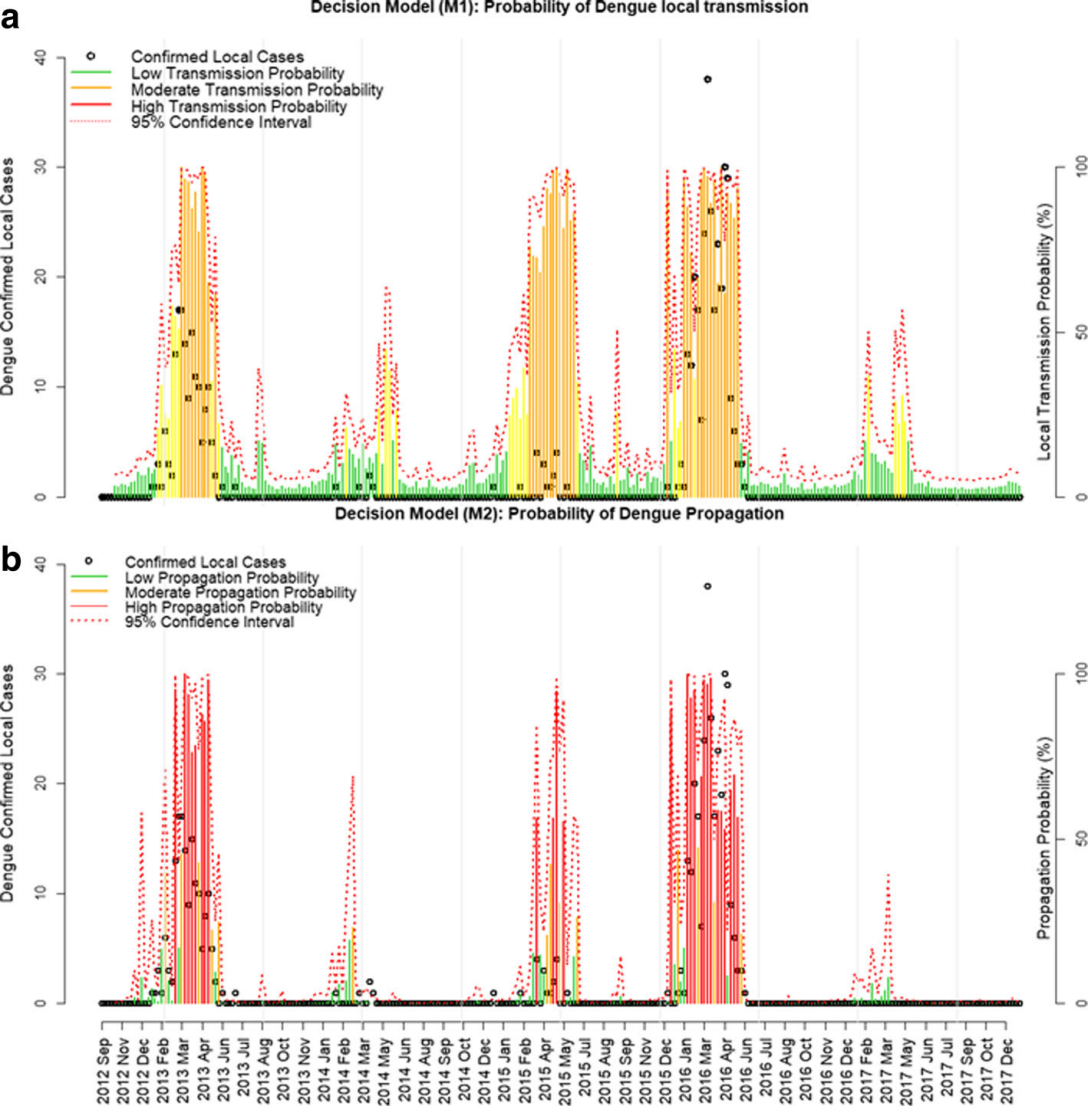
Validation and risk decision model. Probability risk of local dengue transmission initiation (**a**) and propagation (**b**)

We evaluated the accuracy of the M1 and M2 models for classifying weeks with high risk of dengue initiation and dengue propagation, using the ROC curve. For the local initiation of transmission model (M1), the probability cut-off points of 0.2 and 0.6 were selected, while 0.2 and 0.5 were selected for the propagation of transmission model (M2) (Table 3). Models M1 and M2 had high accuracy and specificity (true negative rate), but moderate sensitivity (true positive rate). Probabilities resulting from our models were then aggregated in three different levels of risk: low, moderate and high-risk levels (Table 3, Fig. 7). The majority of the confirmed local cases of dengue occurred during the periods where the probability was considered high-risk for both transmission initiation (M1, Fig. 7a) and propagation (M2, Fig. 7b) models. We observed that both risk decision models reflected the seasonal pattern of the disease during the five epidemic periods of our analyses.

**Table 3:** Validation and decision model for the risk of dengue local transmission introduction (M1) and propagation (M2). Both models were divided into three risk levels (low, moderate and high) for the decision making based on these probabilities

Model	Variable	Time lag (weeks)	AUC	Probability cut-point	Accuracy (%)	Sensitivity TPR (%)	Specificity TNR (%)	Risk decision model		
M1: Local transmission initiation	Dengue imported	3	0.92					< 0.20	Green	Low
	Tweets			0.20	85.22	82.46	86.13	0.20 ≤ x < 0.60	Yellow	Moderate
	Mosquito IMFA			0.60	87.39	61.40	95.95	≥ 0.60	Orange	High
M2: Disease propagation	Dengue imported	4	0.98					< 0.20	Green	Low
	DENV in mosquitoes			0.20	92.61	92.86	92.57	0.20 ≤ x < 0.50	Orange	Moderate
	Tweets			0.50	94.78	75.00	97.52	≥ 0.50	Red	High
	Maximum temperature									

*Abbreviations*: *AUC* area under the curve, *TNR* true negative rate, *TPR* true positive rate, *IMFA* mean infestation of female *Aedes aegypti*, *DENV* dengue virus

## Discussion

We developed a useful probabilistic model for understanding dengue dynamics using risk factors associated with vulnerability and receptivity. The combination of factors that can represent the risk of dengue transmission to be introduced, and then established, is essential for optimized disease modelling and prevention. Vulnerability factors, measured by the number of imported dengue cases and detection of virus circulation in mosquitoes, together with receptivity factors, measured by the abundance of mosquito population and temperature, proved to be good predictors of local transmission of disease. We also observed that the inclusion of a social media variable (tweets with dengue content) increased the predictive estimation capacity of the models. We applied the models to three different moments of the epidemic cycle (beginning, peak and at times of no transmission) and could successfully observe the changing in risk of disease during the epidemic periods. These models could be used to derive risk levels for informing decision-making in temperate areas with risk of dengue occurrence.

In Porto Alegre, imported cases of dengue occurred mostly clustered in the central area of the city, probably associated with the higher social condition of this population that facilitates more frequent travelling. These areas also have a strong capacity to attract visitors, due to the presence of universities, industry and medical facilities [15]. Imported cases, which preceded local transmitted cases, arrived during the epidemic period in other regions of the country [14]. While areas with the highest vector infestation are determined by general sanitation conditions and urbanization, income may be the risk factor for the population exposed to the virus when traveling to other states [15]. Accordingly, DENV circulation in mosquitoes was mostly detected during the transmission period, with the exception of one occurrence (in 2016), possibly associated with one case observed in the same period. In this study, we confirmed the susceptibility status of the Porto Alegre population [17]. The reproductive number *R*_*t*_ was found to exceed the epidemic threshold only during the epidemic seasons of 2013 and 2016 [38], which had a significant number of imported cases and higher local transmission, associated with the epidemic transmission period in other regions of the country [14]. Nevertheless, the incidence of the disease in the area can still be considered a minor burden, with the highest cumulative annual incidence of 21 local cases per 100,000 inhabitants. The WHO suggests that an incidence above 300 may be considered as an epidemic [2].

We divided probability of dengue transmission in two different outcomes: initiation of transmission (Model M1) and propagation (Model M2). Risk factors were evaluated by their influence on initiation of the transmission of local dengue cases (Model M1) in a logistic model to explain the occurrence of one or more local cases in a week. Prevention methods, such as vaccines and mosquito control measures [5], may affect these probabilities, but these are outside this study approach. The selected model that estimates the probability of initiation of local dengue transmission (M1) considered dengue imported cases, mosquito IMFA and tweets, as covariates with a lag of three weeks. While it is difficult to define uniform variables applicable in every context, probable cases were found to be predictive for occurrence of increased risk of dengue transmission and dengue outbreaks [39], since imported cases can initiate local epidemics when appropriate weather conditions are present [40]. The probability of dengue occurrence was also found to increase when the number of adult mosquitoes increased, where 98% of all dengue cases occurred at times of high adult *Ae. aegypti* abundance [26]. Twitter activity was greater in years with high dengue incidence and was demonstrated to be strongly associated with dengue cases, and able to estimate present and future cases [27, 28].

We were curious about the development of dengue transmission, after its initiation in the study area. Risk factors were then evaluated for their influence in the propagation of local cases of dengue. The number of suspected dengue cases, together with meteorological variables, were found to be useful in predicting the occurrence of dengue outbreaks or indicate increased risk of dengue transmission [39]. Seasonal fluctuations of dengue are associated with the irregular circulation of the four DENV serotypes [41, 42]. Interestingly, we observed that detecting DENV in mosquitoes by itself is a significant predictor of local transmission of dengue. However, when considered in combination with other factors, this predictor loses significance, probably because of the sampling method and the reduced amount of data collected during the period. Temperature is also a known predictive factor for dengue incidence, as it affects the rate of virus replication and transmission by mosquitoes, as well as mosquito development and biting rates [43]. During dengue transmission introduction, minimum temperature determines the suitability of the climate for mosquito proliferation and, therefore, for dengue virus transmission [26, 43]. On the other hand, when propagating the disease, maximum temperature will determine the optimal climate for mosquito and virus proliferation, survival and spread [41–43].

Considering both transmission initiation (M1) and propagation (M2) models, we estimated the probability of dengue occurrence in different moments of the transmission period. The summer months (January to March) show higher local transmission probabilities even with a reduced number of mosquitoes, reinforcing the need to keep the mosquito population reduced [17, 19, 25]. Our models allowed the classification of weekly disease risk into three different levels (low, moderate and high). We observed a seasonal pattern of the disease, with a low risk in the inter-epidemic periods, and moderate or high risk during epidemic period, according to the exposure variables. The year of 2017 was found to have no evidence of risk for the disease, as it indeed not occurred in Porto Alegre, but for the whole of Brazil, and also in other countries. We may consider the cyclic characteristic of the disease, and the significant reduction of susceptible population for dengue after two years (2015 and 2016) of important epidemic years. Furthermore, Zika and chikungunya epidemics occurred in these same years simultaneously and the impacts of these immune protective cross-reactive responses are still partially understood. Although all suspected cases of febrile viral diseases were confirmed by ELISA laboratory tests, there may be difficulties in the disease notification process, especially through social media (tweets) where people may be confused about the symptoms. These difficulties may impact on data from the point of view of both notification and social media. A limitation of this analysis is that unreported and asymptomatic dengue cases were not evaluated by both our models and may have influenced disease propagation probability, since recent studies have shown these can contribute to transmission [44]. Also, tweets association with dengue cases may also be influenced by the incidence of human development indices and internet access [28].

A classification of transmission risk based in thresholds is important for a better decision-making regarding prevention and control measures, particularly since it can alert health authorities with three or four weeks in advance. Our models had high accuracy and the majority of the dengue cases occurred when the probability was high for both disease transmission initiation and propagation. An integrated and active surveillance system implemented for infectious diseases, including dengue, in non-endemic areas, is not a reality for most regions in the world at risk disease introduction [19]. Previous studies have attempted to calculate the risk of dengue at different temporal and/or spatial scales [10, 12, 14, 19, 27]. Therefore, our experience in Porto Alegre can help in demonstrating the application of dengue vulnerability and receptivity factors to better adjust and implement risk models. Our models help overcome data source limitations and add into the recommendation for the development of the integration of surveillance across geographical frontiers and modeling across scientific disciplines for real-time risk monitoring. Such assessment platforms could strengthen the awareness of global infectious disease threats and be used before, during, and immediately after mass gathering events [6, 7, 12], as it considers human movement [15, 45], urban development and migration [19, 46].

## Conclusions

New risk maps indicate that tropical and subtropical regions are suitable for extended seasonal or year-round transmission of dengue [40]. Most countries in the tropics and subtropics are at risk of dengue transmission, while temperate areas, like most of the USA, are only at risk during a few months of summer (even if the mosquito vector is present) [43]. Here, we evaluated a rich dataset collected in a temperate model city, Porto Alegre, which experienced several episodes of dengue introduction in the last few years monitored by an integrated surveillance and prevention protocol that includes entomological, virological, and active epidemiological components. We demonstrated how vulnerability and receptivity risk factors can be used in optimized manner to estimate local dengue transmission probability. We developed high accuracy probabilistic models to estimate local transmission initiation and propagation, that successfully estimates disease risk in different scenarios and periods of the year, with three and four weeks, respectively, in advance of real occurrence of the disease outcomes. We propose a decision model with three different risk levels, based on probability of disease occurrence, to assist in prevention and control measures planning applicable to temperate regions with risk of dengue introduction.

## Additional files

Additional file 1: **Figure S1. a** Covariate analyses between response variable “confirmed local dengue cases” and: **b** mosquito abundance index (IMFA); **c** dengue virus presence in mosquitoes; **d** minimum temperature; **e** maximum temperature; **f** confirmed imported dengue cases and, **e** tweets with dengue content. Green arrows indicate selected time periods for further analyses. (TIF 262 kb)

Additional file 2: **Figure S2** Pre-epidemic threshold by moving epidemic method. Dashed lines and circle indicate the exact point of the beginning (red) and the end (green) of the epidemic period. (TIF 256 kb)

Additional file 3: **Table S1.** Total confirmed cases per neighborhood and year. (XLSX 13 kb)

Additional file 4: **Table S2.** Confirmed cases detailed information. (XLSX 38 kb)

Additional file 5: **Figure S3.** Influence of risk factors on the probability of local transmission of dengue. Each variable was evaluated in 5 different time points of forecast (lag: zero to four). **a** Occurrence of each variable during periods with (1) or without (0) dengue local transmission. **b** Probability of dengue local transmission based on each variable selected. (TIF 265 kb)

Additional file 6: **Table S3.** Influence of individual factors on the probability of local dengue transmission (1 or more dengue confirmed cases). Each variable was evaluated in 5 different time points of forecast (lag: zero to four). The odds column indicates how each variable affect the chance of dengue local transmission. (XLSX 14 kb)

Additional file 7: **Figure S4.** Influence of risk factors on the probability dengue propagation. Each variable was evaluated in 5 different time points of forecast (lag: zero to four). **a** Occurrence of each variable during periods with (1) or without (0) dengue local propagation. **b** Probability of dengue local propagation based on each variable selected. (TIF 240 kb)

Additional file 8: **Table S4.** Influence of risk factors on the probability of local dengue propagation (5 or more dengue confirmed cases). Each variable was evaluated in 5 different time points of forecast (lag: zero to four). The odds column indicates how each variable affect the chance of dengue local propagation. (XLSX 12 kb)

## Abbreviations

AIC: Akaike information criterion
DENVs: Dengue virus
IgG: Immunoglobulin type G
IMFA: Mean female *Ae. aegypti* abundance
M1: Model for dengue transmission initiation
M2: Model for dengue transmission propagation
MEM: Moving epidemic method
ROC: Receiving operating characteristic
R_t_: Reproduction numbers
TNR: True negative rate
TPR: True positive rate
