# Supplementary figures and images for: Probability of dengue transmission and propagation in a non-endemic temperate area: conceptual model and decision risk levels for early alert, prevention and control
Source: Parasit Vectors. 2019 Jan 16;12:38. doi: 10.1186/s13071-018-3280-z (PMC6335707; doi:10.1186/s13071-018-3280-z)

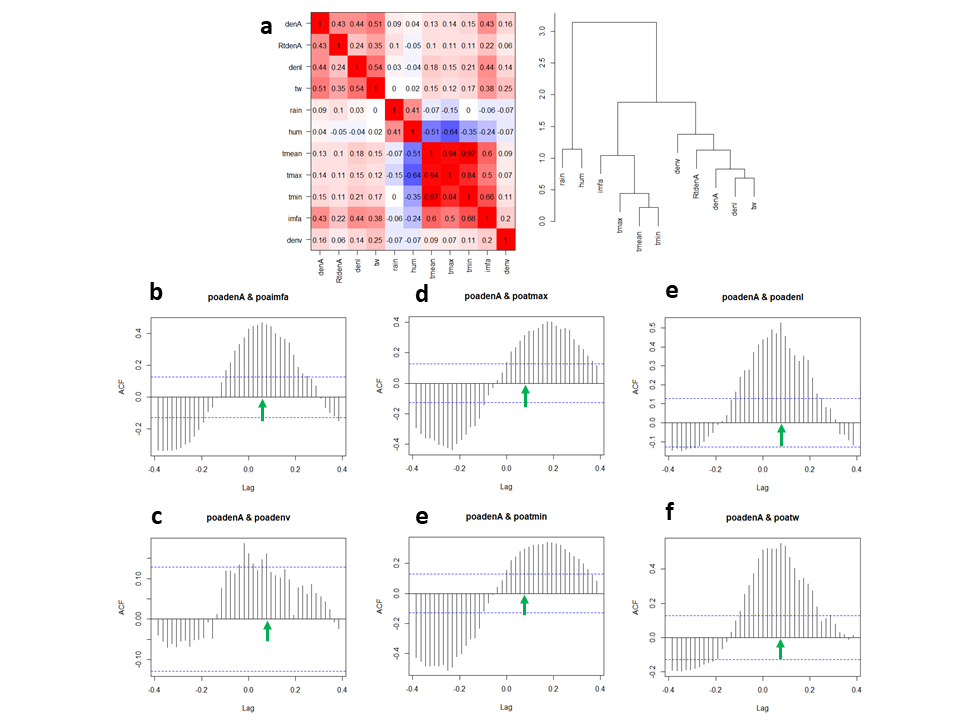

Supplement: Supplementary file 1 — Figure S1. a Covariate analyses between response variable “confirmed local dengue cases” and: b mosquito abundance index (IMFA); c dengue virus presence in mosquitoes; d minimum temperature; e maximum temperature; f confirmed imported dengue cases and, e tweets with dengue content. Green arrows indicate selected time periods for further analyses. (TIF 262 kb) [file 13071_2018_3280_MOESM1_ESM.tif]

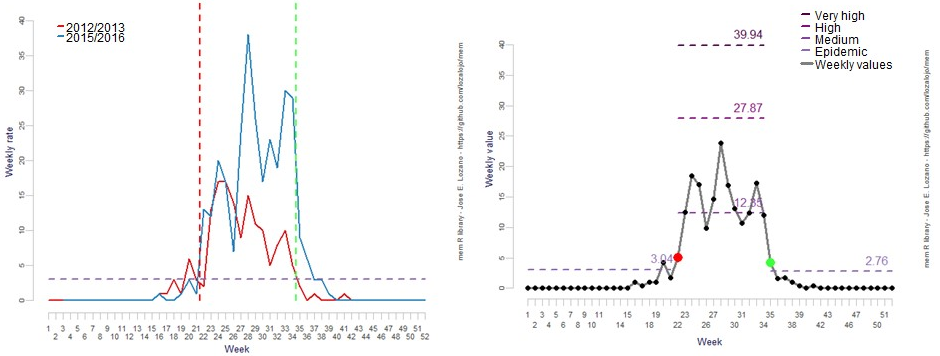

Supplement: Supplementary file 2 — Figure S2 Pre-epidemic threshold by moving epidemic method. Dashed lines and circle indicate the exact point of the beginning (red) and the end (green) of the epidemic period. (TIF 256 kb) [file 13071_2018_3280_MOESM2_ESM.tif]

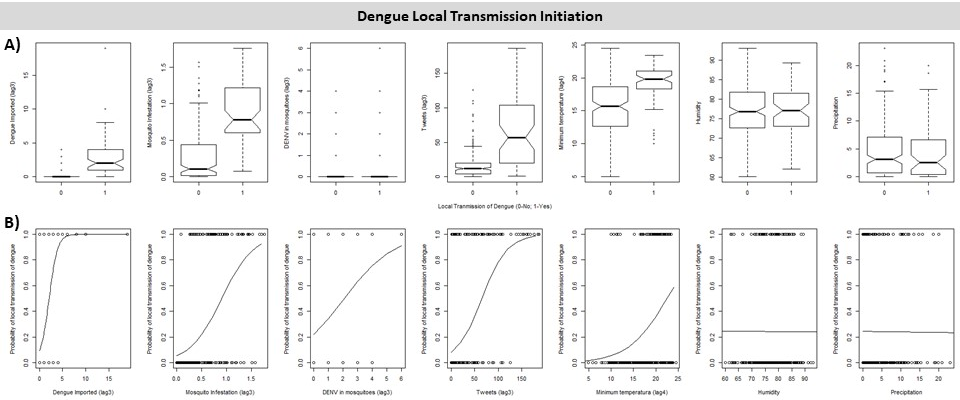

Supplement: Supplementary file 5 — Figure S3. Influence of risk factors on the probability of local transmission of dengue. Each variable was evaluated in 5 different time points of forecast (lag: zero to four). a Occurrence of each variable during periods with (1) or without (0) dengue local transmission. b Probability of dengue local transmission based on each variable selected. (TIF 265 kb) [file 13071_2018_3280_MOESM5_ESM.tif]

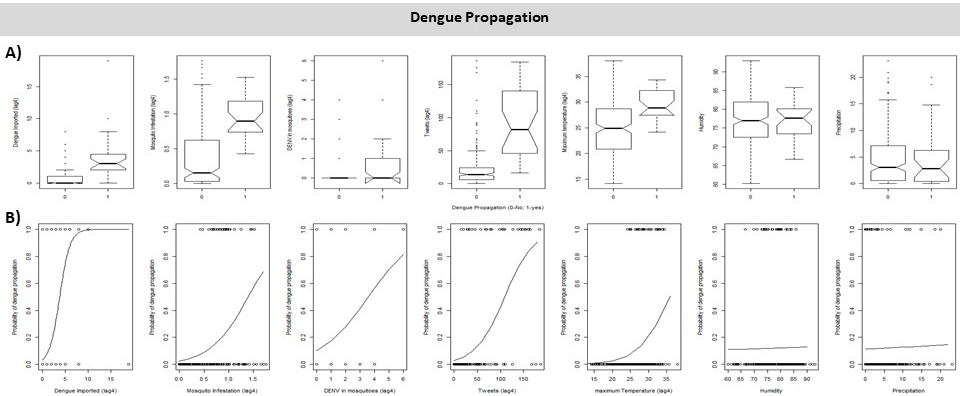

Supplement: Supplementary file 7 — Figure S4. Influence of risk factors on the probability dengue propagation. Each variable was evaluated in 5 different time points of forecast (lag: zero to four). a Occurrence of each variable during periods with (1) or without (0) dengue local propagation. b Probability of dengue local propagation based on each variable selected. (TIF 240 kb) [file 13071_2018_3280_MOESM7_ESM.tif]
